# Swimming performance, maximum O_2_ consumption, EPOC, post-exercise recovery and tissue metabolites after fatigue by *U*_crit_ versus chase protocols in mahi-mahi (*Coryphaena hippurus*), a high-performance pelagic teleost

**DOI:** 10.1242/jeb.251301

**Published:** 2026-04-13

**Authors:** Rachael M. Heuer, Chris M. Wood, John D. Stieglitz, LeeAnn Frank, Daniel Benetti, Martin Grosell

**Affiliations:** ^1^ University of Miami Rosenstiel School of Marine, Atmospheric, and Earth Science, Miami, FL 331491-1031, USA; ^2^ Department of Zoology, University of British Columbia, Vancouver, BC, Canada, V6T 1Z4; ^3^ Department of Biology, McMaster University, Hamilton, ON, Canada, L8S 4K1

**Keywords:** Metabolic rate, Excess post-exercise oxygen consumption, Aerobic metabolism, Anaerobic metabolism, Cost of transport, Lactate, Glycogen

## Abstract

Carangiform fishes are highly active pelagic teleosts, but there have been relatively few studies of their swimming physiology. Recent evidence in other species suggests that swimming to exhaustion using a critical swimming speed (*U*_crit_) protocol may yield higher maximum O_2_ consumption rates (*Ṁ*_O_2_,max_) than chase methodologies. However, there has been little work comparing the internal physiological disturbances and recovery processes resulting from the two methods. We compared these two protocols in young adult mahi-mahi (∼254 g, 26°C, 32 ppt). *Ṁ*_O_2_,max_ after chasing (20 min) was 30% lower than during *U*_crit_ swimming (20-min speed increments). Absolute and factorial aerobic scopes were 42% and 30% lower, respectively, by chasing. Post-exercise recovery was much slower in chased fish, with a ≥1.8-fold higher excess post-exercise O_2_ consumption (EPOC) than in *U*_crit_ fish. Sixty percent of the total O_2_ cost of swimming to fatigue in the *U*_crit_ protocol was incurred during swimming (i.e. extra *Ṁ*_O_2__ above resting O_2_ consumption rate, *Ṁ*_O_2_,rest_) and 40% during recovery (i.e. EPOC). Tissue-specific profiles of metabolites were very different between the two protocols, with the *U*_crit_ treatment causing greater lactate accumulation in red muscle, and chasing causing greater lactate accumulation in the liver and intracellular pH depression in both the red and white muscle at exhaustion, with other differences during recovery. Overall, the results suggest a much greater anaerobic contribution to exercise metabolism caused by chasing. The two protocols yield vastly different results, reflecting different processes. We conclude that the *U*_crit_ protocol provides a much better profile of aerobic capacity.

## Teaser

**Summary:** Maximum oxygen consumption is much greater with a critical swimming speed protocol, whereas excess post-exercise oxygen consumption is much greater with a chasing protocol.

## INTRODUCTION

Pelagic carangiform fishes are elite athletes of the poikilothermic fish world, with swimming performance comparable to that of endothermic tunas (Graham and Dickson, 2004), but their underlying exercise physiology has been investigated only sparsely (Brill, 1996; Graham and Dickson, 2004). However, recent studies on exercise in the mahi-mahi (*Coryphaena hippurus*) using swim-tunnel respirometers have started to correct this information deficit (Mager et al., 2014, 2018; Stieglitz et al., 2016, 2018; Heuer et al., 2021; McGuigan et al., 2021). These investigations have used protocols based on the pioneering work of Brett (1964) with Pacific salmon, in which the fish is subjected to stepwise increases in swimming speed, together with simultaneous measurements of oxygen uptake rate (*Ṁ*_O_2__). The step at which the fish fails due to exhaustion allows the calculation of critical sustainable swimming speed (*U*_crit_). Regression analysis of the relationship between the logarithm of *Ṁ*_O_2__ and swimming speed provides an estimate of the *Ṁ*_O_2__ at *U*_crit_, often termed *Ṁ*_O_2_,max_. Such *U*_crit_ protocols require costly equipment, so an alternative method for achieving exhaustion and estimating *Ṁ*_O_2_,max_ has been adopted by many researchers, although it has never been tried previously with mahi-mahi. This method was developed originally to mimic the effects of angling stress on fish (e.g. Black, 1955; Wood et al., 1983), and then later adopted for respirometric analyses (e.g. Scarabello et al., 1991; Reidy et al., 1995). In this protocol, the fish is manually chased to exhaustion either inside or outside a respirometer, and then the *Ṁ*_O_2__ measurements are started immediately. Extrapolation back to the time of fatigue provides an estimate of *Ṁ*_O_2_,max_.

Over almost 30 years, there have been a number of studies examining whether these two very different methods yield comparable estimates of *Ṁ*_O_2_,max_ (e.g. Reidy et al., 1995; Sylvestre et al., 2007; Norin and Clark, 2016; Killen et al., 2017; Hvas and Oppedal, 2019; Zhang and Gilbert, 2017; Zhang et al., 2020; Rees et al., 2024). Several synthetic reviews have been written on the topic. Norin and Clarke (2016) concluded that the *U*_crit_ method was more reliable for species that are good sustained swimmers, whereas manual chasing to exhaustion was best for species that do not naturally swim for prolonged periods. An extensive review by Killen et al. (2017) found no overall difference between estimates of *Ṁ*_O_2_,max_ between the two methods, regardless of the lifestyle of the species. However, there are exceptions. For example, in some benthic ambush predators, peak oxygen uptake is observed with digestion or combined digestion and exercise rather than exercise alone (Fernandes et al., 2024). Notably, swim performance did not differ between unfed and satiated states in a prior study on mahi-mahi (Stieglitz et al., 2018). Generally, the most recent evidence suggests that the *U*_crit_ protocol consistently provided higher estimates of *Ṁ*_O_2_,max_ than the chasing protocol in most species, when the same individual fish were tested by both methods (Rees et al., 2024).

Curiously, there has been much less interest in comparing the internal physiological changes that occur with these two very different exercise treatments or the metabolic profiles during recovery from them. Given the fact that the *U*_crit_ protocol is intended to measure maximum O_2_ usage rate by contraction of the swimming muscles, whereas the chase protocol is intended to measure maximum O_2_ usage rate by the processes that replenish internal O_2_ and phosphagen stores and metabolize lactate, we might expect rather different responses in systemic physiology during exercise and recovery. We might also expect that the total excess post-exercise oxygen consumption (EPOC; Scarabello et al., 1991; Lee et al., 2003a; Zhang et al., 2018) by the fish during post-fatigue recovery would differ between the two protocols.

Post-chase blood and tissue changes have been relatively well described, at least in salmonids (Wood, 1991; Wang et al., 1994a; Milligan, 1996; Kieffer, 2000), but there is much less information on post-*U*_crit_ blood and tissue changes in any group of fish. Exceptions include the studies of Gallaugher et al. (2001) on chinook salmon (*Oncorhynchus tshawytscha*), Cech et al. (2004) on coho salmon (*Oncorhynchus kisutch*) and Peake and Farrell (2004) on smallmouth bass (*Micropterus dolomieu*).

Against this background, the overarching objective of our study was to compare *U*_crit_ and chase protocols in mahi-mahi to examine whether they would differentially affect estimates of *Ṁ*_O_2_,max_, EPOC, internal physiological state at fatigue and tissue-specific metabolic profiles during recovery. An additional goal was to understand the partitioning of aerobic versus anaerobic costs during the *U*_crit_ protocol. The present paper focuses on *Ṁ*_O_2_,max_, EPOC, and metabolites in red muscle, white muscle and liver.

## MATERIALS AND METHODS

### Experimental animals

Mahi-mahi [*Coryphaena hippurus* (Linnaeus 1758)] were angled by hook and line off the coast of Miami, FL, USA, and brought to the University of Miami Experimental Hatchery (UMEH) to serve as broodstock (Stieglitz et al., 2017). Offspring from this broodstock were reared in a single batch to the young adult life stage (age=3–4 months; mass=253.6±6.7 g, fork length=28.5±0.3 cm, total length=32.7±0.3 cm, *n*=56) using established UMEH techniques. Briefly, mahi-mahi were raised from embryos and fed live planktonic prey during early life stages (enriched rotifers and *Artemia nauplii*) and then transferred to a pelletized diet (Otohime, Reed Mariculture, Campbell, CA, USA) and fed to satiation twice daily. Fish were raised in flow-through seawater and held at 26°C at a salinity of 32–35 ppt for 1 month prior to experimentation. All experiments were performed at 26°C and 32 ppt, and fish were fasted for 24 h prior to experimentation. All animal care and use were carried out in compliance with guidelines provided by the University of Miami's Institutional Animal Care and Use Committee (IACUC protocols 15-019 and 15-067).

### Experimental treatments

Experiments were performed over a period of 40 days in February and March. Prior to experimentation, all mahi-mahi were placed in a 90-liter swim tunnel respirometer (Loligo Systems, Viborg, Denmark) at a speed of 1 body length per second (BL s^−1^) and permitted to acclimate to the swim tunnel for 6 to 12 h prior to experimentation. A speed of 1 BL s^−1^ has been commonly used in prior experimentation with mahi-mahi because these fish are facultative ram ventilators (Stieglitz et al., 2016, 2018) that swim continuously in culture and in the wild. Video recording of the holding tanks revealed a mean routine velocity of 1.7 BL s^−1^ (McGuigan et al., 2021), whereas tagging of wild fish produced a conservative estimate of 0.9 BL s^−1^ on a daily basis (Schlenker et al., 2022). Individuals of this species do not survive if confined in a respirometer without flow. Swim tunnel respirometers were covered so that mahi-mahi could not see observers during acclimation, swimming tests or during recovery.

Mahi-mahi were randomly assigned to one of the six following protocols (*n*=8–10 fish each), which were applied in random order. (1) Control: mahi-mahi were sampled for analysis directly from the swim respirometer with no exercise treatment following 6 to 12 h acclimation. (2) *U*_crit_ fatigue: mahi-mahi were subjected to a *U*_crit_ protocol, where swim speed was incrementally increased by 0.5 BL s^−1^ in 20-min steps until the point of fatigue and were then sampled for analysis. (3) *U*_crit_ recovery 4 h: mahi-mahi were subjected to the *U*_crit_ fatigue protocol, immediately returned to 1 BL s^−1^ to recover, and sampled 4 h after the fatigue time. (4) Chase fatigue: mahi-mahi were removed from the respirometer and chased to exhaustion using a yard stick for 20 min in a 1000-l round tank (diameter=120 cm, depth=90 cm), at which time they were sampled for analysis. (5) Chase recovery 4 h: mahi-mahi were subjected to the chase fatigue protocol, then quickly placed back in the swim tunnel respirometer and allowed to recover at 1 BL s^−1^ for 4 h prior to sampling. And (6) chase recovery 7 h: mahi-mahi were subjected to the chase fatigue protocol, then quickly placed back in the swim tunnel respirometer and allowed to recover at 1 BL s^−1^ for 7 h prior to sampling. This treatment group was added early during the study once it became apparent that 4 h was insufficient to observe full recovery of *Ṁ*_O_2__ in the chase recovery 4 h treatment group.

### Respirometry protocols

Mahi-mahi in all six experimental treatments were first placed in 90-liter Brett-style swim tunnel respirometers (Loligo Systems) for acclimation and assessment of resting *Ṁ*_O_2__ (*Ṁ*_O_2_,rest_). The exact volume of each respirometer was measured and used in all calculations. Control of intermittent respirometry was achieved using AutoResp 2.1.0 software (Loligo Systems). Regardless of treatment, total intervals were in 1200 s (20 min) loops that included a flush period, where the respirometer was open to incoming flow, a stabilization (‘wait’) period, and a measurement period, where the respirometer was closed. Oxygen consumption rates were automatically calculated during the measurement cycle using AutoResp. The wait period was always 30 s, but durations of the measurement and flush periods varied depending on the size of the fish, and the level of exercise. These cycles were varied with the goal of obtaining maximum resolution for *Ṁ*_O_2__ measurements while preventing air saturation from dropping below 80%. *P*_O_2__ was measured using a Pt100 fiber-optic mini-sensor probe that was connected to a Witrox 1 oxygen meter (PreSens Precision Sensing GmbH, Regensberg, Germany). This optode was placed immediately in front of the honeycomb grid at the front of the swim tunnel, and therefore was anterior to the fish. A two-point calibration was performed as in Stieglitz et al. (2016), using UV-sterilized, 100% air-saturated seawater bubbled with an air-stone as the high calibration point and 10 g l^−1^ Na_2_SO_3_ solution as the zero-saturation calibration point. An anemometer with a 30-mm cylinder probe vane wheel flow sensor (Höntzsch GmbH, Waiblingen, Germany) was used to calibrate velocity by measuring speed (cm s^−1^) at different motor output settings (Hz). Temperature was maintained in the tunnels at 26°C using a heater attached to a thermostat (Digital Heat Controller HC-810 mol l^−1^ with 800 W titanium heater, Finnex, Countryside, IL, USA). The respirometer was continuously fed with flow-through fresh, 1-μm filtered, UV-sterilized seawater. This resulted in a ‘blank’ *Ṁ*_O_2__ that was undetectable.

To obtain oxygen consumption rates, the slope of the linear regression line of *P*_O_2__ over time was calculated automatically using AutoResp Software.

### Measurement of resting metabolic rate (*Ṁ*_O_2_,rest_) in all trials

Preliminary trials showed that 6 h of acclimation was sufficient to alleviate the elevation of *Ṁ*_O_2__ associated with handling stress from introduction to the swim tunnel. However, in most cases, mahi-mahi were typically acclimated overnight (∼12 h) to the tunnel and tested in their respective experimental treatments the following day. The mean of the lowest six values collected during this period was used to estimate *Ṁ*_O_2_,rest_ at 1 BL s^−1^, except in those cases where the acclimation period was only 6 h, where the mean of the lowest three values was used. Notably, *Ṁ*_O_2_,rest_ is distinct from standard *Ṁ*_O_2__ (*Ṁ*_O_2_,standard_), which is an extrapolated value that could only be calculated in the fish subjected to the *U*_crit_ protocols (see below).

### Critical swimming speed (*U*_crit_) protocol

*U*_crit_ protocols were performed as previously described for mahi-mahi (Stieglitz et al., 2016; Heuer et al., 2021). Following the acclimation period at 1 BL s^−1^, speed was incrementally increased by 0.5 BL s^−1^ over 20-min steps until the fish was fatigued. Fatigue was defined as continuous brushing with the tail against the screen at the posterior end of the swim tunnel, prolonged resting (30 s) on the back screen, or by the fish losing its place in the water column and becoming pinned against the back screen (Mager et al., 2014; Stieglitz et al., 2016; Heuer et al., 2021).

Immediately following fatigue, mahi-mahi assigned to the *U*_crit_ fatigue treatment were quickly returned to 1 BL s^−1^ so they could be rapidly removed from the respirometer for sampling. Fish assigned to the *U*_crit_ recovery 4 h treatment were quickly returned to 1 BL s^−1^ and remained at this speed in the respirometer for 4 h after fatigue, at which time, they were sampled.

*U*_crit_ was calculated using the following equation (Brett, 1964):
(1)

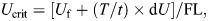
where *U*_f_ (cm s^−1^) is the highest swimming velocity achieved during a complete interval, *T* is the length of time spent at the final, highest swim velocity, *t* is the set time for the step interval, d*U* (cm s^−1^) is the increment in swim speed of each step, and FL is the fish fork length (cm). Using AutoResp Software, a solid blocking correction was applied to address differences in water velocity owing to the size of the mahi-mahi (Bell and Terhune, 1970).

### Measurement of standard metabolic rate (*Ṁ*_O_2_,standard_) and *Ṁ*_O_2_,max_ in the *U*_crit_ trials

To determine *Ṁ*_O_2_,standard_ and *Ṁ*_O_2_,max_ in the *U*_crit_ trials, the logarithm of *Ṁ*_O_2__ (mg O_2_ kg^−1^ h^−1^) was plotted as a function of swimming speed (BL s^−1^) on a linear scale, and a least squares linear regression was performed for each fish (Mager et al., 2014; Stieglitz et al., 2016; Heuer et al., 2021). From these data, *Ṁ*_O_2_,standard_ was calculated using the *y*-intercept and *Ṁ*_O_2_,max_ was the *Ṁ*_O_2__ at *U*_crit_ predicted by the regression. As the fish used in the chase trials (see below) were swum at only one speed (1.0 BL s^−1^) prior to their fatigue treatment, their *Ṁ*_O_2_,standard_ could not be calculated. Therefore, *Ṁ*_O_2_,rest_ at 1 BL s^−1^ was used rather than *Ṁ*_O_2_,standard_ for calculations of aerobic scope and EPOC both for this reason, and because *Ṁ*_O_2_,standard_ is not a meaningful reference point for a ram-ventilating species that is swimming continually both in the wild (Schlenker et al., 2022) and in the swim tunnel. Accordingly, absolute aerobic scope was calculated as *Ṁ*_O_2_,max_−*Ṁ*_O_2_,rest_, and factorial aerobic scope was calculated as *Ṁ*_O_2_,max_/*Ṁ*_O_2_,rest_.

The aerobic cost of transport (ACOT, mg O_2_ kg^−1^ m^−1^) for each individual fish at each speed up to *U*_crit_ was calculated in the traditional manner by dividing the *Ṁ*_O_2__ by the swimming speed. The incremental cost of swimming (ICOT, mg O_2_ kg^−1^ m^−1^) at each speed up to *U*_crit_ was calculated for each fish by dividing *Ṁ*_O_2__−*Ṁ*_O_2_,rest_ by the swimming speed, as described by Lee et al. (2023a), though these authors called it ‘net cost of transport’. The total aerobic cost of swimming to *U*_crit_ (total oxygen cost to *U*_crit_, TOC*_U_*_crit;_ mg O_2_ kg^−1^) for each individual fish was also calculated in a similar manner to that used by Lee et al. (2023a), though we used direct measurements, rather than the modeling approach employed by those authors. In brief, the total O_2_ used above *Ṁ*_O_2_,rest_ was calculated for each 20-min step increment above 1.0 BL s^−1^ as (*Ṁ*_O_2__−*Ṁ*_O_2_,rest_)×0.333, where 0.333 represents a fraction of an hour. To yield TOC*_U_*_crit_, the values for each step were then summed and added to the O_2_ consumed above *Ṁ*_O_2_,rest_ in the final fatigue step calculated as (*Ṁ*_O_2_,max_−*Ṁ*_O_2_,rest_)×*X*, where *X* represents the fatigue time as a fraction of an hour. TOC*_U_*__crit_,_ values (aerobic cost of swimming, mg O_2_ kg^−1^) could then be compared with EPOC values (anaerobic cost of swimming, mg O_2_ kg^−1^), the latter calculated as outlined below.

### Chase to fatigue protocol

Mahi-mahi were removed from the swim tunnel respirometer immediately after the final acclimation measurement cycle and quickly moved to an aerated circular chase tank (1000 liters) containing UV-sterilized, 1-μm filtered seawater held at 26°C. Following an introduction to this tank, mahi-mahi were chased manually using a yard stick by two experimenters taking turns for 20 min, a time period found to induce full exhaustion in pilot research. By full exhaustion, we mean that there was no burst swimming at all, though the fish was still able to swim slowly around the tank. At this point, they were killed for sampling if they were in the chase fatigue treatment.

For chase recovery 4 h and 7 h treatments*,* the exhausted mahi-mahi were immediately placed back into the swim tunnel respirometer at 1.0 BL s^−1^. Because some fish were introduced back into the respirometer during the flush cycle, and some were reintroduced during the measurement cycle, a calculation based on time after introduction and a back-extrapolation from EPOC analysis to fatigue time (see below) was deemed the most accurate reflection of *Ṁ*_O_2_,max_.

### Measurement of *Ṁ*_O_2_,max_ in the chase recovery trials

For chase recovery 4 h and 7 h treatments, decay functions were used to predict *Ṁ*_O_2_,max_ at the end of chasing, taken as the fatigue point (time zero). The *Ṁ*_O_2__ values during recovery were plotted (mg O_2_ kg^−1^ h^−1^, *y*-axis) over time (h, *x*-axis), and in most cases, four-parameter double exponential decay functions were fitted to the curves. In three cases (chase recovery 4 h fish), three-parameter single exponential decay functions were used instead as they provided better fits (higher *R*^2^) to the data. Back-extrapolation of these functions to the time of fatigue provided estimates of *Ṁ*_O_2_,max_. These same plots were also used for EPOC measurements (see below).

### Measurement of EPOC in the *U*_crit_ recovery and chase recovery trials

In the *U*_crit_ recovery 4 h group, EPOC values (mg O_2_ kg^−1^) were estimated from similar four-parameter double exponential decay functions fitted to the *Ṁ*_O_2__ values during recovery from fatigue. In two cases, three-parameter single exponential decay functions were used instead as they provided higher *R*^2^ values. In the chase recovery 4 h and 7 h groups, comparable plots were used to estimate EPOC values. In all three groups, after adding these fits, a horizontal line representing the pre-exercise *Ṁ*_O_2_,rest_ in the same fish was added. To assess EPOC, integration (*Ṁ*_O_2__×time) under the fitted line and above the horizontal line was performed using ImageJ (National Institutes of Health, Bethesda, MD, USA). The area was bounded on the left by the time at which *U*_crit_ occurred or chase ended (i.e. fatigue time for both), and on the right either by the time of measurement (4 or 7 h) or by the time at which the fitted curve intercepted the horizontal *Ṁ*_O_2_,rest_ line, whichever came first. Thus, in the *U*_crit_ treatments, the *Ṁ*_O_2_,max_ calculated at *U*_crit_ was used as the time zero data point in assessing EPOC. For chase treatments, the estimated *Ṁ*_O_2_,max_ at the end of chasing was used as the time zero data point.

The second metric used to assess recovery was the mg O_2_ kg^−1^ h^−1^ value above *Ṁ*_O_2_,rest_ at recovery at the final time point for each respective group. This value was calculated based on the decay function at either 4 or 7 h, depending on the treatment group. Representative traces have been provided in the Results.

### Allometric analyses

To explore whether *Ṁ*_O_2__, TOC*_U_*_crit_ and EPOC data should be scaled allometrically, the logarithms of the individual values per fish within a treatment group were regressed against the logarithms of individual fish mass, with intercept (*a*) and slope (*b*), the latter representing the allometric mass scaling coefficient. *R*^2^ values were assessed for significance.

### Sampling protocols

For sampling, fish were removed from the respirometer or the chase tank (for the chase fatigue group) and immediately placed into an ice-cold bath (4°C) of aerated seawater containing a lethal dose of MS-222 (Syndel, Parksville, BC, Canada) (1 g l^−1^ neutralized to pH 8.2 using NaOH, 4°C). First, a caudal blood sample was taken by caudal puncture (<1 min), followed by tissue sampling (<3 min). Tissues were harvested in the following order: spleen, white muscle, red muscle and then liver. The spleen and blood samples were used in another study. White muscle was taken from the epaxial muscle mass in the midsection of the fish, and red muscle was obtained near the lateral line in the same region. White muscle, red muscle and liver were immediately freeze-clamped between two cryoblocks pre-frozen in liquid nitrogen, wrapped in foil, placed into liquid nitrogen and stored in an ultra-cold freezer (−80°C) for later analyses.

### Analytical methods for tissue metabolites and intracellular pH (pH_i_)

Samples were removed from the ultracold freezer, and extraneous skin and bloody parts were cut off. The frozen tissues were then ground to a fine powder under liquid N_2_ using a ceramic mortar and pestle system (Cryogrinder, OPS Diagnostics, NJ, USA). The powder was aliquoted into two different ice-cold buffers. The first was used for the determination of pH_i_ and contained 150 mmol l^−1^ potassium fluoride (KF) and 6 mmol l^−1^ of the disodium salt of nitriloacetic acid (both from Sigma-Aldrich, USA) at pH 6.8 (Pörtner et al., 1990). The second was for the determination of lactate, glycogen, glucose and free glucosyl units, and contained 60 mmol l^−1^ sodium fluoride (NaF) in a 25 mmol l^−1^ sodium citrate buffer set to pH 4.2. In both cases, the powder was weighed frozen and remained frozen until it entered the buffer.

For pH_i_ determination by the method of Pörtner et al. (1990), approximately 200 mg of powder was added to 200 µl of ice-cold buffer in a 500-µl microcentrifuge tube, then topped with buffer, stirred briefly with a needle to release bubbles, then sealed, vortexed for a few seconds, and centrifuged for 15 s at 5000 ***g***. The pH of the supernatant (representing pH_i_) was then measured in triplicate at 26°C using a combination glass pH micro-electrode (Accumet, Fisher Scientific), meter (ThermoFisher Orion Star A221) and IAPUC calibration buffers (Radiometer Analytical-Copenhagen, Demmark).

For metabolites, approximately 50 mg of powder was added to 1 ml of ice-cold buffer in a 2-ml microcentrifuge tube, then homogenized in an ice bath for 60 s using an IKA™ 210 Ultra-Turrex tissue homogenizer (Werke GMBH & Co., Staufen, Germany) at medium speed. The tubes were then centrifuged at 10,000 ***g*** for 5 min at 4°C. The supernatant was decanted as 200-µl aliquots, which were then frozen in liquid N_2_ and stored at −80°C until later assay. Lactate was measured directly on the supernatant using a Sigma-Aldrich MAK329 L-lactate kit. For tissue assays, standards were made up in the NaF-sodium citrate buffer. Glycogen, total glucosyl units and free glucosyl units were measured on a separate stored aliquot of supernatant using an Enzychrom™ E2GN-100 glycogen assay kit (Bioassay Systems, Hayward, CA, USA). The assay is based on the measurement of glucosyl units in the presence (total) and absence (free glucosyl units) of an enzyme complex that breaks down glycogen to glucose, the difference representing the glycogen content. Free glucosyl units are mainly glucose but may include a small fraction of 6C intermediates. However, for the sake of convenience, free glucosyl units are referred to as ‘glucose’ in the Results. For red and white muscle, the assays were performed directly on the supernatant; however, for liver, the supernatant was further diluted 20-fold with the NaF-sodium citrate buffer prior to assay. Standards were made up in the same buffer, and the colored product was read at 570 nm. The standards were supplied as glycogen weights in mg, which we converted to glucosyl units in mmol, assuming a molecular weight of 162 for 1 glucosyl unit when it is present in glycogen.

### Statistical analyses

One-way ANOVAs were used to compare means across treatment groups, followed by the Tukey's *post hoc* test to identify significant differences among treatment groups where the sample sizes (*n*) for each group were similar. In some cases, log transformations were performed to meet assumptions of normality and homogeneity of variance. In other cases, the Kruskal–Wallis ANOVA on ranks was used where these criteria were not achieved. In comparisons among performance metrics at different swimming speeds, sample sizes decreased at higher speeds, so the Holm–Šidák test was used to provide greater power. Pearson's correlation analysis was used to explore relationships between various measured parameters, and regression lines were fitted by the method of least squares. Analysis of covariance (ANCOVA) was used to compare the slopes of allometric regression equations. Statistical significance for all tests was accepted at *P*<0.05, and data have been reported as means±s.e.m. (*n*), where *n* is the number of fish, with individual data points shown in the figures.

Assessments of *Ṁ*_O_2_,rest_, *Ṁ*_O_2_,max_, aerobic scope, TOC*_U_*_crit_ and EPOC across common treatments were pooled, where appropriate, after first checking that there were no significant differences between the treatment groups. Thus, chase fatigue, chase recovery 4 h and chase recovery 7 h represented one set of common treatments, whereas *U*_crit_ and *U*_crit_ recovery 4 h formed the other common treatment. In these instances, chase and *U*_crit_ treatment groups were compared using Student's two-tailed *t*-tests, or Mann–Whitney tests instances where assumptions of normality could not be met. Paired Student's *t*-tests were used to evaluate whether changes were significant within the same fish, and one-sample Student's *t*-tests were employed to test whether means were significantly different from a specific value.

## RESULTS

### Oxygen consumption and swimming performance

**Fig. 1.**
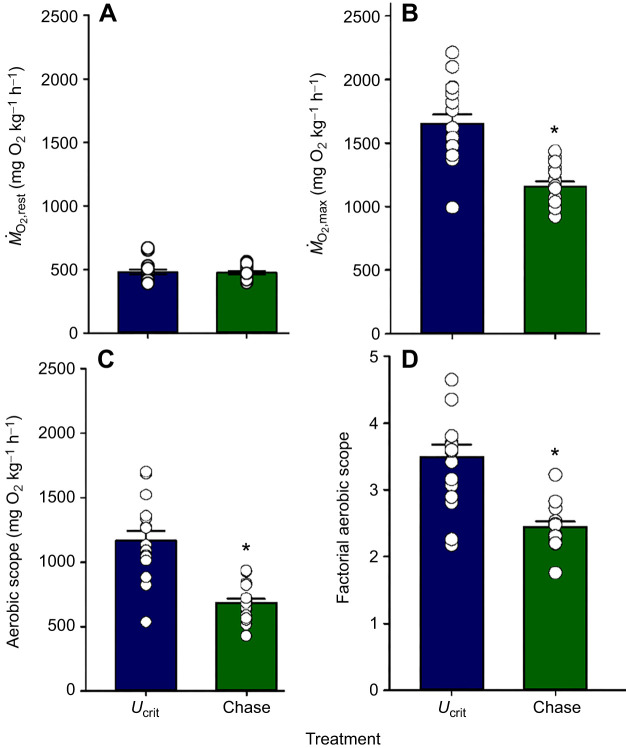
**Metrics of aerobic metabolic rate in mahi-mahi.** (A) Resting O_2_ consumption rate (*Ṁ*_O_2_,rest_), (B) maximum O_2_ consumption rate (*Ṁ*_O_2_,max_), (C) absolute aerobic scope and (D) factorial aerobic scope in fish swimming at 1.0 BL s^−1^ that were subsequently used in *U*_crit_ (*n*=17) or chase (*n*=17) protocols. Data are means±1 s.e.m. with individual data points shown. Asterisks indicate significant differences at *P*<0.05. Full statistical results are reported in Table S2.

*Ṁ*_O_2_,standard_ did not differ (Mann–Whitney test, *U*=25, *P*=0.312) between the two *U*_crit_ treatment groups, averaging 317±25 mg* *kg^−1^ h^−1^ (*n*=17) overall. *Ṁ*_O_2_,standard_ was not determined in the chase groups, because they had not been put through the *U*_crit_ protocol needed to estimate this value by regression extrapolation. Instead, they had been swum at only one speed (1.0 BL s^−1^) prior to chasing, so as to provide a measurement of their *Ṁ*_O_2_,rest_ value. *Ṁ*_O_2_,rest_ was therefore measured in every treatment group, and did not differ (Kruskal–Wallis ANOVA, *H*=0.18, d.f.=5, *P*=0.999) among those fish used in the two *U*_crit_ treatments, those used in the three chase treatments, or those used in the control treatment, averaging 480±11 mg* *kg^−1^ h^−1^ (*n*=51) overall (Fig. 1A). *Ṁ*_O_2_,rest_ was greater (Mann–Whitney test, *U*=78, *P*<0.001) than *Ṁ*_O_2_,standard_ and served as the reference value used in all subsequent calculations.

**Fig. 2.**
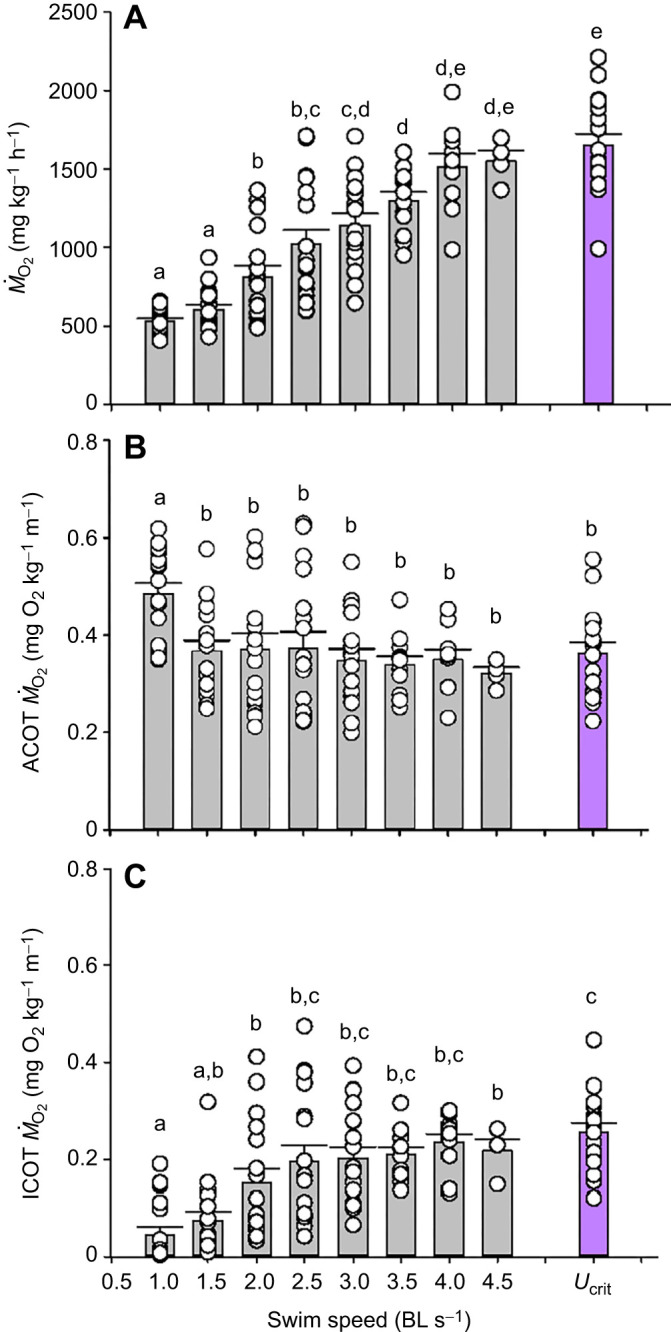
**Metrics of swimming performance in mahi-mahi swum at increasing speeds (20-min steps) in the *U*_crit_ protocol.** (A) *Ṁ*_O_2__ at each step (ANOVA, *F*_8,118_=39.88, *P*<0.0001). (B) Aerobic cost of transport (ACOT) at each step (*F*_8,118_=2.680, *P*=0. 0096). (C) Incremental aerobic cost of transport (ICOT) at each step (*F*_8,118_=9.502, *P*<0.0001). Data are means±1 s.e.m. with individual data points shown; *n*=17 at 1.0, 1.5, 2.0 and 2.5 BL s^−1^, *n*=16 at 3.0 BL s^−1^, *n*=12 at 3.5 BL s^−1^, *n*=10 at 4.0 BL s^−1^, *n*=4 at 4.5 BL s^−1^, and *n*=17 at *U*_crit_ (3.76±0.14 BL s^−1^). Means sharing the same letter are not significantly different from one another at *P*<0.05 by the Holm–Šidák test. Full statistical results are reported in Table S2.

Mean *Ṁ*_O_2__ (ANOVA, *F*_8,118_=39.88, *P*<0.0001) increased progressively with swimming speed in the first four steps of the *U*_crit_ trials (Fig. 2A). Above 3.0 BL s^−1^, there were no further increases (Holm–Šidák tests, *t*=≤1.303, *n*=17–4, *P*≥0.1325) in mean *Ṁ*_O_2__ up to *U*_crit_ as more and more fish started to fail, and *n* became sequentially lower. These exhausted fish therefore no longer contributed to the mean for that swimming speed. Inspection of the data showed that these were generally the fish with the higher *Ṁ*_O_2__ values at the preceding speed. Their loss from the means made the overall relationship appear more linear, whereas it was closer to logarithmic on an individual fish basis.

The aerobic cost of transport (ACOT, the *Ṁ*_O_2__ per unit swimming speed) (ANOVA, *F*_8,118_=2.680, *P*=0.0096) declined from 1.0 to 1.5 BL s^−1^ (Holm–Šidák test, *t*=3.268, *n*=17, *P*=0.0459) and thereafter exhibited no change (*t*=≤0.731, *n*=17–4, *P*≥0.9999) up to *U*_crit_ (Fig. 2B). The incremental aerobic cost of swimming (ICOT, the elevation of *Ṁ*_O_2__ above *Ṁ*_O_2_,rest_ per unit swimming speed) (ANOVA, *F*_8,118_=9.502, *P*<0.0001) increased from 1.0 to 2.0 BL s^−1^ (Holm–Šidák test, *t*=3.346, *n*=17, *P*=0.0276) and thereafter did not change (*t*≤1.916, *n*=17–4, *P*≥0.6957) up to *U*_crit_ (Fig. 2C).

*U*_crit_ did not differ (Student's two-tailed *t*-test, *t*=−1.502, d.f.=15, *P*=0.154) between the two *U*_crit_ treatment groups, averaging 3.76±0.14 BL s^−1^ (*n*=17) overall. *Ṁ*_O_2_,max_, as estimated by these *U*_crit_ treatments, was 1652±74 mg* *kg^−1^ h^−1^ (*n*=17), whereas *Ṁ*_O_2_,max_ as estimated by the two chase treatments was 30% lower (Mann–Whitney test, *U*=16, *P*<0.001) at 1158±39 mg* *kg^−1^ h^−1^ (*n*=16) (Fig. 1B). As a result, the calculated aerobic scope was 42% lower (Mann–Whitney test, *U*=20, *P*<0.001) by the chase protocol (*n*=16) relative to the *U*_crit_ protocol (*n*=17), and the factorial aerobic scope was 30% lower (*U*=32, *P*<0.001) in the chase treatments (Fig. 1C,D). To check whether there was any systematic bias in estimating *Ṁ*_O_2_,max_ by back-extrapolation from EPOC data in the chase trials versus from forward-extrapolation from the *Ṁ*_O_2__ data during the swimming trials, we calculated *Ṁ*_O_2_,max_ by both methods for the fish in the *U*_crit_ recovery 4 h treatment group. The *Ṁ*_O_2_,max_ values were only 1.0±1.3% (*n*=9) lower by back-extrapolation from the EPOC data, a negligible difference (Student's one-sample *t*-test, *t*=0.7932, d.f.=8, *P*=0.4505).

**Fig. 3.**
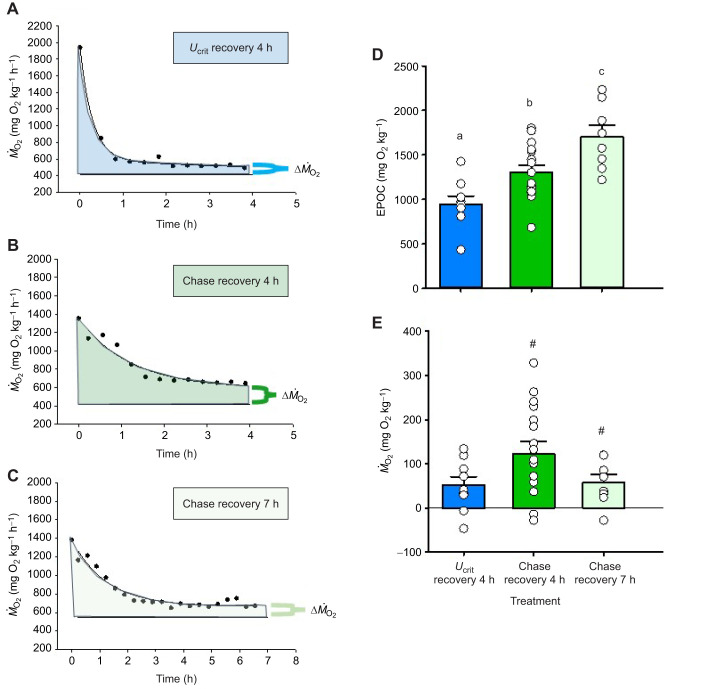
**Metrics of excess post-exercise oxygen consumption (EPOC) in mahi-mahi during recovery from fatigue.** (A–C) Typical recordings of *Ṁ*_O_2__ over time in a fish in the (A) *U*_crit_ recovery 4 h, (B) chase recovery 4 h and (C) *U*_crit_ recovery 7 h treatments. The left vertical line represents time 0 h (the estimated time at which recovery started) and the right vertical line represents the time at which the EPOC measurement ended, the horizontal line represents *Ṁ*_O_2_,rest_ in the particular fish, the bracket represents the elevation in *Ṁ*_O_2__ (i.e. Δ*Ṁ*_O_2__) persisting at the end of the EPOC measurement, and the shaded area represents the integrated EPOC value. (D) EPOC values in the three treatments (ANOVA, *F*_2,32_=12.344, *P*<0. 001). (E) Elevations of *Ṁ*_O_2__ above *Ṁ*_O_2_,rest_ (i.e. Δ*Ṁ*_O_2__) at the end of the EPOC measurement in the three treatments (Kruskal–Wallis ANOVA, *H*=3.934, d.f.=2, *P*=0.140). In D and E, data are means±1 s.e.m., with *n*=9 in the *U*_crit_ recovery 4 h treatment, *n*=16 in the chase recovery 4 h treatment and *n*=8 in the *U*_crit_ recovery 7 h treatment. Means sharing the same letter are not significantly different from one another at *P*<0.05. In E, the symbol # indicates that the elevated *Ṁ*_O_2__ in a treatment group was significantly different from the *Ṁ*_O_2_,rest_ in these same fish (*P*<0.05). Full statistical results are reported in Table S2.

Comparison of mean EPOC values (ANOVA, *F*_2,30_=12.344, *P*<0. 001) revealed that mean EPOC in chased fish by 4 h recovery (1307±77 mg kg^−1^, *n*=16) was 38% higher (Holm–Šidák test, *t*=2.779, *n*=16, *P*=0.009) than that in *U*_crit_ treatment (944±66 mg kg^−1^, *n*=9) measured at the same time (Fig. 3D). When the EPOC measurements were extended to 7 h in the chased fish (1701±0.130 mg kg^−1^, *n*=8), the elevation increased to 80% (Holm–Šidák test, *t*=4.968, *n*=9, *P*<0.001) (Fig. 3D).

The mean absolute elevations in *Ṁ*_O_2__ above *Ṁ*_O_2_,rest_ in the three groups are compared in Fig. 3E (Kruskal–Wallis ANOVA, *H*=3.934, d.f.=2, *P*=0.140). The mean *Ṁ*_O_2__ value measured at 4 h in the *U*_crit_ recovery treatment was no longer elevated above *Ṁ*_O_2_,rest_ in these same fish (Student's one-sample *t*-test, *t*=1.389, d.f.=8, *P*=0.225) (Fig. 3D). Mean *Ṁ*_O_2__ values in the chase recovery 4 h treatment, and at the 4 h mark in the chase recovery 7 h treatment, were very similar to one another and were therefore combined. At 4 h recovery, the mean elevation above *Ṁ*_O_2_,rest_ in these chased fish averaged 28±6% (*n*=16) (Student's one-sample *t*-test, *t*=4.940, d.f.=16, *P*=0.0002). At 7 h recovery, the *Ṁ*_O_2__ in the chased animals still remained elevated above *Ṁ*_O_2_,rest_ (*t*=3.821, d.f.=7, *P*=0.0065) in the same individuals by 13±4% (*n*=9) (Fig. 3E).

The total oxygen cost of swimming to *U*_crit_ (TOC*_U_*_crit_) in the *U*_crit_ recovery 4 h treatment was 1420±168 mg O_2_ (*n*=9), substantially greater (paired Student's two-tailed *t*-test, *t*=2.673, d.f.=8, *P*=0.0282) than the 4 h EPOC value of 944±91 mg O_2_ (*n*=9) in these same fish.

As EPOC was statistically complete by 4 h in fish swum to fatigue by the *U*_crit_ protocol, the total O_2_ cost of swimming to *U*_crit_ and recovering was the sum of TOC*_U_*_crit_ (1420 mg kg^−1^) and 4 h EPOC (944 mg kg^−1^), amounting to 2364±202 mg kg^−1^ (*n*=9). Thus 60% of the cost was incurred during the exercise phase (which lasted 80–190 min), and 40% during the recovery phase (240 min). We could not calculate the O_2_ cost of the exercise phase (20 min) or the total cost for fish swum to fatigue by the chase protocol, but at least 1701±0.130 mg kg^−1^ (*n*=8) was incurred during the recovery phase (7 h EPOC) that was still ongoing at 420 min after exhaustion.

None of the values reported above (Figs 1–3) were allometrically scaled. Within the individual treatments, there were relationships at *P*<0.05 between log mass (*X*, g) and log *Y* (mg O_2_ fish^−1^ h^−1^ for *Ṁ*_O_2__ values or mg O_2_ fish^−1^ for EPOC and TOC*_U_*_crit_ values) for all parameters except EPOC values at *U*_crit_ recovery 4 h (*P*=0.164) (Table S1). For *Ṁ*_O_2__ values, the allometric scaling coefficients yielded by these regressions ranged from 0.665 (for *Ṁ*_O_2_,max_ by *U*_crit_) to 0.896 (for *Ṁ*_O_2_,standard_). There were higher coefficients for EPOC and TOC*_U_*_crit_ values, ranging from 0.945 (for EPOC at *U*_crit_ recovery 4 h) to 1.533 (for TOC*_U_*_crit_ in the *U*_crit_ recovery 4 h treatment). As none of the scaling coefficients except TOC*_U_*_crit_ were different from one another by ANCOVA or from 1.0 at *P*<0.05, we deemed it inappropriate to use different coefficients for different treatments given this wide variation and the relatively narrow absolute range of body masses (159–387 g). To assess the possible error associated with this decision, we compared the mean unscaled values reported above with the values predicted by the appropriate allometric regression equations for a mahi-mahi weighing 254 g, the mean mass for the experimental population. As illustrated in Table S1, the differences were negligible, reflecting potential overestimates of 1–5% for the various parameters.

### Tissue metabolites and pH_i_

#### Red muscle

**Fig. 4.**
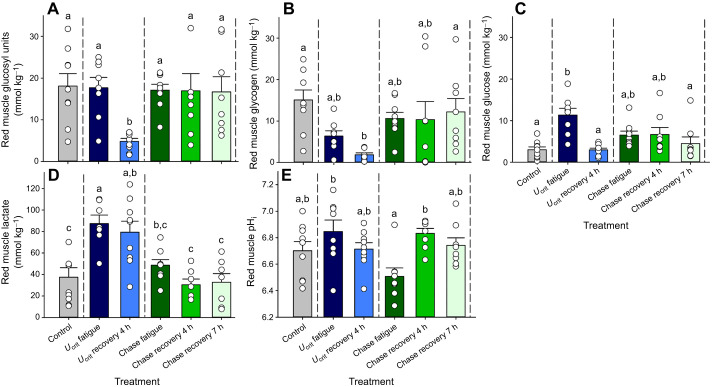
**Metabolites in the red muscle of mahi-mahi in control, exercise and recovery treatments.** (A) Total glucosyl units (ANOVA, *F*_5,45_=3.729, *P*=0.0066), (B) glycogen (*F*_5,45_=3.716, *P*=0.0067), (C) glucose (includes other 6C intermediates; *F*_5,45_=6.623, *P*<0.0001), (D) lactate (*F*_5,41_=8.887, *P*<0.0001) and (E) intracellular pH (pH_i_) (*F*_5,44_=3.689, *P*=0.0071). Data are means±1 s.e.m. (*n*=7–9) with individual data points shown. Means sharing the same letter are not significantly different from one another at *P*<0.05. Full statistical results are reported in Table S2.

Treatment-related variations occurred in red muscle total glucosyl units (ANOVA, *F*_5,45_=3.729, *P*=0.0066), glycogen (*F*_5,45_=3.716, *P*=0.0067), glucose (*F*_5,45_=6.623, *P*<0.0001), lactate (*F*_5,41_=8.887, *P*<0.0001) and pH_i_ (*F*_5,44_=3.689, *P*=0.0071) (Fig. 4).

Under resting conditions in mahi-mahi swimming at 1 BL s^−1^ after 6–12 h in the swim tunnel, the total concentration of glucosyl units was approximately 18 mmol kg^−1^, of which approximately 75% was glycogen and 25% was glucose (Fig. 4A–C). Immediately after *U*_crit_ fatigue, total glucosyl units remained unchanged (Tukey's test, *q*=2.528, *n*=8,9, *P*=0.4841), but glycogen now accounted for only 36% of the total glucosyl units, whereas the glucose concentration had increased (*q*=6.875, *n*=8,9, *P*=0.0002) to 64% of the total glucosyl units (Fig. 4A–C). However, at *U*_crit_ recovery 4 h, total glucosyl units in red muscle were severely reduced (*q*=6.232, *n*=9,9, *P*=0.0009) to only about 5 mmol kg^−1^, of which 2 mmol kg^−1^ was glycogen and 3 mmol kg^−1^ was glucose (Fig. 4A–C). Lactate concentration in red muscle at rest was approximately 37 mmol kg^−1^, and this increased (*q*=6.127, *n*=9,7, *P*=0.0012) to approximately 87 mmol kg^−1^ at *U*_crit_ fatigue and remained elevated (79 mmol kg^−1^) at *U*_crit_ recovery 4 h (*q*=5.496, *n*=9,9, *P*=0.0012) (Fig. 4D).

The chase protocols elicited a very different pattern of response, inasmuch as there was no depletion of total glucosyl units in the red muscle at any time, with concentrations remaining unchanged from resting levels at chase fatigue, chase recovery 4 h and chase recovery 7 h (Tukey's tests, *q*≤3.092, *n*=9,8,8, *P*≥0.2646; Fig. 4A). Glycogen was better preserved throughout, representing approximately 60–70% of the total at all three sampling times, with glucose accounting for 30–40% (Fig. 4B,C). The lactate profile was also very different in the red muscle of the chased fish. The mean lactate concentration (48 mmol kg^−1^) at chase fatigue was similar to the control value (37 mmol kg^−1^) and much lower (*q*=4.629, *n*=8,7, *P*=0.0246) than at *U*_crit_ fatigue (87 mmol kg^−1^), with a return to resting levels (*q*≤0.8519, *n*=9,7,7, *P*>0.9903) at chase recovery 4 h and chase recovery 7 h (Fig. 4D).

Red muscle pH_i_ was approximately 6.70 in resting mahi-mahi (Fig. 4E). Responses to the two exercise protocols were again very different: mean pH_i_ increased by approximately 0.18 units at *U*_crit_ fatigue (Tukey's test, *q*=2.251, *n*=9,8, *P*=0.5631) but fell by approximately 0.2 units at chase fatigue (*q*=3.129, *n*=9.8, *P*=0.2527). As a result, the two fatigue values were substantially different (*q*=5.326, *n*=8,8, *P*=0.0061) from each other (Fig. 4E). At *U*_crit_ recovery 4 h, pH_i_ was back to control levels (*q*=0.2226, *n*=9,9, *P*>0.9999), whereas at chase recovery 4 h, red muscle pH_i_ had rebounded from the chase fatigue acidosis (*q*=5.110, *n*=8,8, *P*=0.0094) to a level comparable to that at *U*_crit_ fatigue (*q*=2.135, *n*=8,8, *P*=0.6600). Control levels were restored at chase recovery 7 h (*q*=0.6637, *n*=9,8, *P*=0.9970) (Fig. 4E).

#### White muscle

**Fig. 5.**
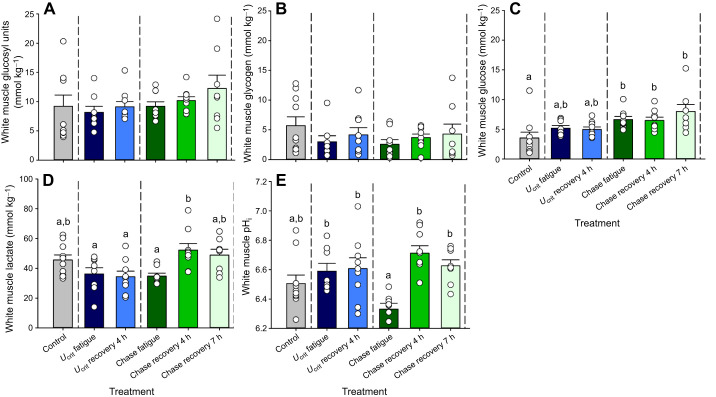
**Metabolites in the white muscle of mahi-mahi in control, exercise and recovery treatments.** (A) Total glucosyl units (ANOVA, *F*_5,47_=0.9904, *P*=4338), (B) glycogen (*F*_5,47_=0.9917, *P*=0.4817), (C) glucose (includes other 6C intermediates; *F*_5,47_=4.449, *P*=0.0021), (D) lactate (*F*_5,46_=4.480, *P*=0.0022) and (E) pH_i_ (*F*_5,46_=5.403, *P*=0.0005). Data are means±1 s.e.m. (*n*=8-10) with individual data points shown. Means sharing the same letter are not significantly different from one another at *P*<0.05. Full statistical results are reported in Table S2.

Treatment-related variations did not occur in white muscle total glucosyl units (ANOVA, *F*_5,47_=0.9904, *P*=4338) or in glycogen (*F*_5,47_=0.9917, *P*=0.4817; Fig. 5A,B). However, responses were seen in white muscle glucose (*F*_5,47_=4.449, *P*=0.0021), lactate (*F*_5,46_=4.480, *P*=0.0022) and pH_i_ (*F*_5,46_=5.403, *P*=0.0005; Fig. 5C–E).

Under resting conditions, the control concentration of total glucosyl units in white muscle was approximately 9 mmol kg^−1^ (Fig. 5A), only about half of that in red muscle (cf. Fig. 4A). This was composed of approximately 62% glycogen and 38% glucose (Fig. 5B,C). As in red muscle (cf. Fig. 4A), immediately after *U*_crit_ fatigue, total glucosyl units remained unchanged (Fig. 5A), with glucose tending to rise at the expense of glycogen. However, unlike the situation in red muscle at *U*_crit_ recovery 4 h, the total concentration of glucosyl units remained stable at this time, and the relative contributions of glycogen (46%) versus glucose (54%) did not change (Fig. 5B,C). Lactate concentration in white muscle at rest was approximately 45 mmol kg^−1^ (Fig. 5D), very similar to that in red muscle (cf. Fig. 4D). However, unlike red muscle, lactate levels in white muscle remained unchanged in fish sampled at *U*_crit_ fatigue and at *U*_crit_ recovery 4 h (Fig. 5D).

Total glucosyl units at chase fatigue, chase recovery 4 h and chase recovery 7 h remained constant in white muscle (Fig. 5A). However, the glucose component increased relative to the resting control value at all three sampling times – chase fatigue, chase recovery 4 h and chase recovery 7 h (Tukey's tests, *q*≥4.097, *n*=9,9,8, *P*≤0.0597) (Fig. 5C). The glucose component accounted for approximately 70% of the total at all three times such that the glycogen fraction was reduced to approximately 30% (Fig. 5B). The lactate profile also differed from that of red muscle after fatigue by chasing. Lactate concentrations in white muscle fell slightly from 46 mmol kg^−1^ at rest to 35 mmol kg^−1^ at chase fatigue (*q*=2.977, *n*=10,8, *P*=0.3028), but then rebounded to approximately 52 mmol kg^−1^ at chase recovery 4 h (*q*=4.673, *n*=8.9, *P*=0.0214), with no further change at chase recovery 7 h (Fig. 5D). Lactate concentration was higher at chase recovery 4 h than at *U*_crit_ recovery 4 h (*q*=4.673, *n*=9,9, *P*=0.0134).

White muscle pH_i_ was approximately 6.5 (Fig. 5E), lower than the red muscle pH_i_ of 6.7 (cf. Fig. 4E) in mahi-mahi at rest. At *U*_crit_ fatigue and *U*_crit_ recovery 4 h, white muscle pH_i_ did not change. At chase fatigue, white muscle pH_i_ was approximately 6.3, much lower (Tukey's test, *q*=4.478, *n*=8,8, *P*=0.0308) than in the *U*_crit_ fatigue treatment (approximately 6.6) (Fig. 5E) or in the red muscle at this time (cf. Fig. 4E). This was followed by a marked rebound to 6.6–6.7 at both chase recovery 4 h and chase recovery 7 h (*q*≥4.927, *n*=8,9,8, *P*≤0.0132) (Fig. 5E).

#### Liver

**Fig. 6.**
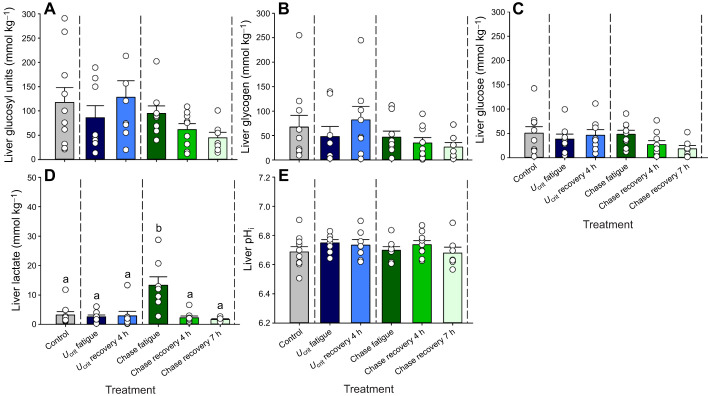
**Metabolites in the liver of mahi-mahi in control, exercise and recovery treatments.** (A) Total glucosyl units (ANOVA, *F*_5,45_=1.885, *P*=0.1173), (B) glycogen (*F*_5,45_=1.089, *P*=0.3794), (C) glucose (includes other 6C intermediates; *F*_5,45_=1.441, *P*=0.2281), (D) lactate (*F*_5,43_=9.378, *P*<0.0001) and (E) pH_i_ (*F*_5,43_=0.8242, *P*=0.5394). Data are means±1 s.e.m. (*n*=7–10) with individual data points shown. Means sharing the same letter are not significantly different from one another at *P*<0.05. Full statistical results are reported in Table S2.

Treatment-related variations did not occur in liver total glucosyl units (ANOVA, *F*_5,45_=1.885, *P*=0.1173), glycogen (*F*_5,45_=1.089, *P*=0.3794), glucose (*F*_5,45_=1.441, *P*=0.2281) or pH_i_ (*F*_5,43_=0.8242, *P*=0.5394; Fig. 6A–C,E). However, treatment effects were seen for liver lactate concentration (*F*_5,43_=9.378, *P*<0.0001; Fig. 6D).

The total concentration of glucosyl units in liver in resting mahi-mahi (∼120 mmol kg^−1^; Fig. 6A) was approximately 5- to 10-fold higher than in red muscle (cf. Fig. 4A) or white muscle (cf. Fig. 5A). However, values were highly variable among individuals and treatment groups, as were the glycogen and glucose components (Fig. 6A–C). In all treatments, the glycogen component averaged 50–65% of the total. As a result of this variability, none of the changes among treatment groups were significant; however, a trend was observed for both glycogen and glucose depletion (Fig. 6B,C) at chase recovery 4 h and especially at chase recovery 7 h. At chase recovery 7 h, the total concentration of glucosyl units was reduced to 44 mmol kg^−1^ (Fig. 6A). Lactate concentrations in the liver were uniform across most treatments at very low levels of 2–5 mmol kg^−1^ (Fig. 6D) in contrast to the 10- to 20-fold higher concentrations in red muscle (cf. Fig. 4D) and white muscle (cf. Fig. 5D). The one exception was a much higher lactate concentration of approximately 13 mmol kg^−1^ in the livers of the chase fatigue group relative to all other treatment groups (Tukey's tests, *q*≥7.206, *n*=7–9, *P*<0.0001) (Fig. 6D).

In light of this variability in other hepatic parameters, liver pH_i_ values were surprisingly constant among individuals, averaging approximately 6.7 overall, with no differences among the treatment groups (Fig. 6E).

## DISCUSSION

### Overview

Overall, the present study provides convincing evidence that *U*_crit_ and chase methodologies used to elicit exhaustive exercise result in very different whole-animal performances, respirometric responses, internal disturbances and metabolic recovery profiles in mahi-mahi. Key differences in mahi-mahi include significantly higher *Ṁ*_O_2_,max_ values by the *U*_crit_ method, and significantly higher and longer-lasting EPOC values by the chase protocol. Our results suggest that red muscle plays a larger role in *U*_crit_ exercise, whereas white muscle plays a larger role in chase exercise. Therefore, *U*_crit_ exercise relies more on aerobic metabolism, whereas chase exercise relies more on anaerobic metabolism.

### Performance in *U*_crit_ trials

The present data (Fig. 2A) compare favorably with those of a previous study (Stieglitz et al., 2016) using mahi-mahi of approximately the same size and age at a slightly higher temperature (28°C versus 26°C). *Ṁ*_O_2_,max_ values (1652 mg* *kg^−1^ h^−1^) were identical in the two studies, whereas *U*_crit_ was 8% lower in the present study (3.76 BL s^−1^), probably attributable to the slight temperature difference. Heuer et al. (2021) reported that the optimal temperature for swimming performance in mahi-mahi is 28°C. Unlike Stieglitz et al. (2016) and McGuigan et al. (2021), we did not see a swimming speed at which ACOT was clearly lowest (i.e. often called the optimal speed). Rather, we recorded ACOT values that were not significantly different from 1.5 BL s^−1^ through to *U*_crit_ (Fig. 2B). Nevertheless, these costs were virtually identical to the minimum ACOT values reported by Stieglitz et al. (2016) and McGuigan et al. (2021) at optimal speeds of 2.0 to 3.0 BL s^−1^. ACOT analyses (often called just COT) can paint a misleading picture of the true cost of the exercise component because they include the contribution of *Ṁ*_O_2_,rest_, which becomes progressively diluted by the true costs of swimming as speed increases. The incremental aerobic cost of swimming (ICOT, the elevation of *Ṁ*_O_2__ above *Ṁ*_O_2_,rest_ per unit swimming speed), is more informative in this regard, as it eliminates the contribution of *Ṁ*_O_2_,rest_. Our ICOT analysis (Fig. 2C) demonstrated that swimming costs per unit speed increased significantly from 1.0 to 2.0 BL s^−1^ and thereafter did not increase at higher speeds up to *U*_crit_. It may well be that although ICOT does not continue to rise at higher speeds, the additional true cost of high performance is met by an increasing contribution from anaerobic metabolism that must be repaid during the recovery period. A very similar pattern was reported in another elite athlete, the sockeye salmon (*Oncorhynchus nerka*), by Lee et al. (2003a). In salmonids, it is well established that anaerobic metabolism by the white muscle is recruited once swimming speed exceeds approximately 70% *U*_crit_ (Burgetz et al., 1998; Richards et al., 2002). If the same occurred in the present mahi-mahi, this would correspond to a speed of approximately 2.5 BL s^−1^.

We are aware of only one other study that has performed an analysis of TOC*U*_crit t_ versus EPOC so as to partition the O_2_ costs of swimming to *U*_crit_ between the exercise and recovery phases. Lee et al. (2003a) reported that at 8–18°C, in three different species of Pacific salmon, all approximately 10-fold larger than the present mahi-mahi, at least 80% of the swimming costs were incurred during the exercise phase, and less than 20% were incurred during the recovery phase. This contrasts with the 60% exercise phase (TOC*_U_*_crit_, aerobic) and 40% recovery phase (EPOC, anaerobic repayment) distribution of O_2_ costs seen in mahi-mahi. The post-*U*_crit_ EPOCs in the mahi-mahi were not only much larger, but also much longer lasting (240 min) than in the salmon (42–78 min). Furthermore, in salmonids of similar size to the present mahi-mahi such as chinook salmon (Gallaugher et al., 2001) and Atlantic salmon (*Salmo salar*) (Hvas and Oppedal, 2019) tested at their optimal temperatures (10–13°C), *Ṁ*_O_2_,max_ was approximately 70% lower whereas *U*_crit_ was only approximately 25% lower than the present values measured on mahi-mahi. The picture that emerges is one of much higher aerobic and anaerobic swimming costs in mahi-mahi during *U*_crit_ tests than in salmonids, but it is debatable whether this is due only to the much higher temperature (e.g. Brett and Glass, 1973; Lee et al., 2003b; Heuer et al., 2021) and/or to differences in the mechanical efficiency of swimming. Other possible contributing factors may include differences in ecology and phylogeny.

### Comparison with other pelagic carangiform fishes

Cobia (*Rachycentron canadum*) of similar size, tested at the same temperature (26°C) as the present mahi-mahi, exhibited 40–50% lower values of *Ṁ*_O_2_,rest_, *Ṁ*_O_2_,max_ and *U*_crit_ (Nelson et al., 2017), though the comparison is confounded by the fact that the cobia were heavily instrumented with a Doppler flow probe in the ventral aorta. Ten-fold larger cobia at 28°C exhibited even lower values of *Ṁ*_O_2_,rest_ and *Ṁ*_O_2_,max_ (Crear et al., 2020). Exercise has been studied more extensively in the carangiform yellowtail kingfish (*Seriola lalandi*) (Clark and Seymour, 2006; Brown et al., 2011; Pan et al., 2020). Again, comparisons are difficult because of instrumentation, lower temperatures (20–23°C), or 3- to 10-fold larger body masses of the fish used in those studies. Nevertheless, *Ṁ*_O_2_,rest_ and *Ṁ*_O_2_,max_ were 50–70% lower and quite uniform, whereas *U*_crit_ values varied from 2.3 to 3.9 BL s^−1^ in a manner that did not appear to be size-dependent in yellowtail kingfish. Overall, the mahi-mahi appears to have higher aerobic performance than these other two species, but additional studies are needed on the large number of other species of pelagic carangiform fishes.

### Performance in chase trials

The fact that it took 20 min of manual chasing to elicit complete exhaustion (of the two experimenters as well as the fish!) is testimony to the exercise capacity and endurance of mahi-mahi. In our experience, comparable fatigue is reached in salmonids in less than 10 min (Wood, 1991). We could not measure *Ṁ*_O_2__ during the chase, but we suspect that exercise during this period was largely fueled by anaerobic metabolism. This is based on the 30% lower *Ṁ*_O_2_,max_ at the end of the chase versus *U*_crit_ treatments (Fig. 1B–D), the many differences in tissue metabolites (Figs 4–6) discussed below, and the longer-lasting and 80% greater EPOC (Fig. 3D,E), which would be sufficient to support more than 3 h of *Ṁ*_O_2__ at rest. This greater size and duration of EPOC after fatigue by chasing versus fatigue by *U*_crit_ has also been seen in salmonids (reviewed by Zhang et al., 2018).

The mahi-mahi is a prized sport fish subject to intense recreational and commercial fishing (Bignami et al., 2014; Merton et al., 2022a,b; Carlson, 2025). The post-chase EPOC alone and its extended duration (>7 h) has implications for catch-and-release angling, a stressor that in many ways is similar to chasing (cf. Wood et al., 1983; Holder et al., 2022). We observed no mortalities up to 7 h post-fatigue, but in future studies it would be interesting to test whether repeated chasing during the recovery period is fatal. Schlenker et al. (2022) captured wild mahi-mahi by angling, tagged them, allowed them to recover in a large tank onboard a ship for 12 h, then released them. Their 8-day survival rate was only 50%; most of the mortalities were probably due to predation, but it is unknown what role handling and captivity played in these events post-release.

### Tissue metabolites after *U*_crit_ fatigue versus chase fatigue

A concern in our measurements is whether the very high resting concentrations of lactate in red muscle (∼37 mmol kg^−1^; Fig. 4D) and white muscle (∼45 mmol kg^−1^; Fig. 5D) are real, or resulted from rapid conversion of glucosyl units to lactate by glycolysis in the time between removal of the fish from the respirometer and freeze-clamping. For example, in salmonids, normal resting lactate concentrations in these tissues are usually much lower, less than 5 mmol kg^−1^ (e.g. Wang et al., 1994a,b; Richards et al., 2002). If glycolytic conversion due to muscle contraction or hypoxaemia during sampling did occur, then measured lactate would have been too high, and total glucosyl units, glycogen and pH_i_ would all likely have been too low at rest, and values at other times may have been differentially impacted. To prevent this, we immersed the fish in a very high concentration of neutralized MS-222 as recommended by Dobson and Hochachka (1987) and Wang et al. (1994b) and additionally presented it at 4°C to these 26°C-acclimated animals. Nevertheless, there were usually a few tail flips before the fish became immobile, though this did not occur in mahi-mahi sampled at fatigue. This phenomenon may have contributed not only to the possible biases in absolute values noted above, but also to the generally greater variability of these parameters in red and white muscle in the control treatment (Figs 4 and 5), thereby making it more difficult to detect differences in the exercise treatments. However, it must be remembered that these are highly active animals swimming at 1.0 BL s^−1^ at high temperature in the resting condition, so high muscle lactate concentrations may be real. Perhaps the best comparison is with another highly active teleost, the skipjack tuna (*Katsuwonis polaris*), studied at a water temperature of 25°C (Arthur et al., 1992). Here, initial resting lactate concentrations in white muscle were approximately 39 mmol kg^−1^, which declined to 12 mmol kg^−1^ at 3 h after the animals were spinally blocked, artificially ventilated and restrained to prevent muscle contraction. Given these uncertainties, we present the red and white muscle data in Figs 4 and 5 at face value with the caveat that they should be interpreted with caution.

Metabolite profiles pointed to a greater reliance on red muscle and aerobic metabolism in supporting exercise in mahi-mahi subjected to the *U*_crit_ fatigue treatment versus the chase fatigue treatment, whereas anaerobic metabolism in white muscle appeared to dominate in the latter. In this regard, our results agreed with those of Peake and Farrell (2004) on smallmouth bass. In the present study on mahi-mahi, the evidence included much greater increases in glucose (Fig. 4C) and especially lactate (Fig. 4D) in red muscle in the *U*_crit_ fatigue treatment versus the chase fatigue treatment. The increase in red muscle lactate amounted to approximately 50 mmol kg^−1^ and persisted at *U*_crit_ recovery 4 h (Fig. 4D). If these lactate elevations had originated endogenously, then a depletion of red muscle glycogen stores by approximately 25 mmol kg^−1^ would have been expected, whereas the total glucosyl units at rest were less than this (Fig. 4A), and the observed glycogen depletion was only approximately 8 mmol kg^−1^ (Fig. 4B). It seems more likely that the lactate originated exogenously, perhaps from adjacent white muscle, and was ‘shuttled’ to red muscle (Brooks, 2018) to serve as a fuel to be burned in aerobic metabolism (Bilinski and Jonas, 1972). Oxidation of lactate in red muscle would also explain the much higher pH_i_ in red muscle in *U*_crit_ fatigue versus chase fatigue treatments (Fig. 4E). There was only a modest, non-significant elevation in lactate accompanying the low pH_i_ in the red muscle of the chase fatigue fish, with a return to resting levels of both parameters during recovery (Fig. 4D,E). Notably, white muscle pH_i_ was also depressed in chase fatigue fish (Fig. 5E), indicative of ‘lactic acid’ production and/or ATP breakdown in this tissue (Mommsen and Hochachka, 1988; Hochachka and Mommsen, 1983; Wang et al., 1994a). As white muscle lactate increased only during recovery (Fig. 4D), the lactate anions produced during chasing exercise were likely rapidly exported to the extracellular fluid. The extracellular space in liver tissue is high, about 15% of hepatic mass in trout (Munger et al., 1991). Therefore, the elevated liver lactate concentration seen only in the chase fatigue fish (13 mmol kg^−1^; Fig. 6E) likely resulted, at least in part, from elevated plasma lactate, though we cannot eliminate the possibility of endogenous lactate production by the liver tissue itself.

### Conclusions

Although a number of studies have examined whether *U*_crit_ fatigue versus chase fatigue protocols provide comparable estimates of *Ṁ*_O_2_,max_ in fish (see Introduction), we conclude that they do not in mahi-mahi. Furthermore, the two procedures have very different effects on the post-exercise recovery processes. Most importantly, *Ṁ*_O_2_,max_ was significantly higher in the *U*_crit_ fatigue treatment than in the chase fatigue treatment, whereas EPOC showed the opposite pattern. We do not find this conclusion surprising, because *U*_crit_ exercise relies more on aerobic metabolism, whereas chase exercise relies more on anaerobic metabolism. We would not expect contraction of the swimming muscles at maximum sustainable swimming speed to necessarily consume O_2_ at the same rate as the processes working at their highest rate to restore internal phosphagen and O_2_ stores and clear the end-products of anaerobic metabolism immediately after exhaustion. Although both approaches yield valuable information on exercise physiology, they are like apples and oranges, and in no way equivalent. Data resulting from them should not be conflated. Our conclusions are in accord with the recent review of Rees et al. (2024). These authors surveyed studies using both methods on a number of species and concluded that the *U*_crit_ protocol consistently provided higher estimates of *Ṁ*_O_2_,max_. Indeed, although we have used the traditional term *Ṁ*_O_2_,max_ in our present studies, we support the recommendation of Rees et al. (2024) that going forward, the two estimates of *Ṁ*_O_2_,max_ should be given different names – ‘peak *Ṁ*_O_2_,swim_’ versus ‘peak *Ṁ*_O_2_,recovery_’. We also hope that going forward, future studies will not just focus on *Ṁ*_O_2_,max_, but will also measure the internal physiological changes and metabolic recovery processes resulting from these very different protocols, as we have done in mahi-mahi.

## Supplementary Material

10.1242/jexbio.251301_sup1 Supplementary information

Table S2. Excel file of statistical results for each Figure panel in the main paper.
