# Supplementary material for: Swimming performance, maximum O2 consumption, EPOC, post-exercise recovery and tissue metabolites after fatigue by Ucrit versus chase protocols in mahi-mahi (Coryphaena hippurus), a high-performance pelagic teleost
Source: J Exp Biol. 2026 Apr 13;229(7):jeb251301. doi: 10.1242/jeb.251301 (PMC13120674; doi:10.1242/jeb.251301)
Supplement: Supplementary information [file jexbio-229-251301-s1.pdf]

Table S1. Allometric scaling relationships for O_2 in mahi-mahi under different conditions. The two right-hand columns compare the allometrically scaled values predicted by the relevant scaling equation for a 254-g fish with the non-scaled group mean value, which would be equivalent to a scaling coefficient of 1.0.

Y (mg O_2 fish^{-1} h^{-1} for $\dot{M}O_2$, mg fish^{-1} for EPOC and TOCU_{crit})	Equation where Y is defined in left-hand column and X is Log body mass (g)	R^2	P	Scaled to 254-g fish by equation (mg O_2 kg^{-1} h^{-1} for $\dot{M}O_2$, mg O_2 kg^{-1} for EPOC and TOCU_{crit})	Non-scaled group mean (mg O_2 kg^{-1} h^{-1}) for $\dot{M}O_2$, mg kg^{-1} for EPOC and TOCU_{crit})
$\dot{M}O_{2standard}$ (N = 17)	LogY = 0.896^{C}*LogX - 0.2667	0.320	0.0180	303	317
$\dot{M}O_{2rest}$ (N = 56)	LogY = 0.770^{C}*LogX +0.2275	0.620	< 0.0001	471	480
$\dot{M}O_{2max}$ in U_{crit} trials (N = 17)	LogY = 0.665^{C}*LogX +0.0190	0.422	0.0048	1633	1652
$\dot{M}O_{2max}$ in *Chase* trials (N =16)	LogY = 0.791^{C}*LogX +0.5609	0.707	< 0.0001	1145	1158
EPOC in U_{crit} *recovery 4h* trial (N = 9)	LogY = $0.^{945CD}$*LogX +0.0882	0.257	0.1639	902	944
EPOC at 4h in *Chase* trials (N=16)	LogY = $1.^{115CD}$*LogX - 0.1706	0.550	0.0010	1276	1307
EPOC at 7h in *Chase* trial (N = 8)	LogY = 1.151^{CD}*LogX - 0.1412	0.727	0.0072	1666	1701
TOCU_{crit} in U_{crit} *recovery 4h* trial (N =9)	LogY = 1.533^{D}*LogX - 1.1455	0.4852	0.0371	1369	1420

Slopes (b values = allometric scaling coefficients) in equations (LogY = slope*Log X + intercept) are not significantly different where b values share the same superscripted capital letter.

Table S2. Excel file of statistical results for each Figure panel in the main paper.

Available for download at
https://journals.biologists.com/jeb/article-lookup/doi/10.1242/jeb.251301#supplementary-data
